# Benefits of SGLT2 inhibitors in patients with diabetes and advanced chronic kidney disease – where have we gone so far?

**DOI:** 10.3389/fcdhc.2025.1759340

**Published:** 2026-01-19

**Authors:** Anila Duni, Paraskevi Tsavourelou, Maria Triantafyllidou, Louiza Gkika, Christos Georgopoulos, Eleni Stamellou, Athanasios Kitsos, Evangelia Dounousi

**Affiliations:** 1 Department of Nephrology, University Hospital of Ioannina, Ioannina, Greece; 2 Department of Nephrology, Faculty of Medicine, School of Health Sciences, University of Ioannina, Ioannina, Greece

**Keywords:** advanced CKD, cardiovascular disease, diabetic kidney disease, hemodialysis, kidney failure, peritoneal dialysis, residual kidney function, SGLT2 inhibitors

## Abstract

SGLT2 inhibitors have transformed the care of patients with diabetes, chronic kidney disease (CKD), and cardiovascular disease. Nevertheless, the efficacy of SGLT2 inhibitors as well as potential associated risks remains to be further clarified in the setting of advanced diabetic kidney disease. Indirect evidence and secondary analyses from the landmark SGLT2 trials as well as emerging data from recent studies including exclusively patients with diabetes and advanced CKD, indicate that the renal and cardiovascular benefits of SGLT2 inhibitors persist even in these patients. Although the use of SGLT2 inhibitors in patients with diabetes undergoing dialysis remains investigational, preliminary evidence from experimental and clinical studies seems promising in terms of multifaceted positive outcomes, including preservation of residual kidney function. Furthermore, the available data until now does not indicate an increase in risk in patients with diabetes and advanced CKD regarding acute impairment of kidney function or other adverse outcomes of interest including diabetic ketoacidosis, infections, fractures risk and amputations. The aim of this review is to present the current knowledge available on the utility of SGLT2 inhibitors in patients with diabetes and advanced CKD so as to provide a foundation for their implementation in clinical practice. Future experimental research shall further elucidate the pleiotropic effects of SGLT2 inhibitors so as to expand their indications in the setting of diabetes and advanced CKD. Finally, the results of ongoing clinical trials in patients with diabetes and kidney failure as well as in dialysis patients are much anticipated.

## Introduction

The burden of chronic kidney disease (CKD) is on the rise with type 2 diabetes being the leading cause (1). Approximately 850 million people are estimated to have CKD worldwide corresponding to 10% of the global population and by 2040, CKD is expected jump to the fifth place as a cause of disability-adjusted life years (DALYs) (2, 3). About type 2 diabetes, the projected prevalence is expected to reach 642 million in 2040 compared to 415 million in 2015 (4). Considering that nearly 40% of patients with type 2 diabetes have CKD, and diabetic nephropathy is responsible for nearly half of all cases of kidney failure requiring renal replacement therapy (RRT) or transplantation, the magnitude of the problem is immense and demands the most rigorous efforts to address it (4, 5). Furthermore, patients with advanced CKD and type 2 diabetes have greater probability of dying from cardiovascular causes, mainly heart failure (HF) and atherosclerotic cardiovascular disease (CVD) than progressing to kidney failure (6–8).

During recent years, randomized controlled trials have demonstrated the efficacy of SGLT2 inhibitors to transform the care of patients with diabetes and kidney disease by significantly retarding eGFR decline, thus delaying kidney failure, as well as by improving cardiovascular outcomes (9, 10). Yet, in patients with severely impaired kidney function, the efficacy of SGLT2 inhibitors in terms of glucosuria and natriuresis is reduced, thus creating a hesitancy with regard to SGLT2 inhibitor implementation in this setting (11). Yet, emerging evidence not only from the landmark SGLT2 inhibitor trials but from novel studies focusing exclusively on patients with CKD stage 4 and 5 as well, have challenged the conception that in this setting any interventions aiming to delay the relentless disease progression to kidney failure requiring dialysis are futile (12). Moreover, improvement of cardiovascular outcomes in patients with advanced CKD and those on dialysis would be of paramount importance, as the risk of adverse cardiovascular events has been shown to be exponentially greater in stage 4 compared to stage 1 CKD (13). Therefore, according to current guidelines, SGLT2 inhibitors initiation is allowed in patients with eGFR above 20 ml/min/1.73 m2 and this treatment may be continued until the start of dialysis (14–16).

**Figure 1:**
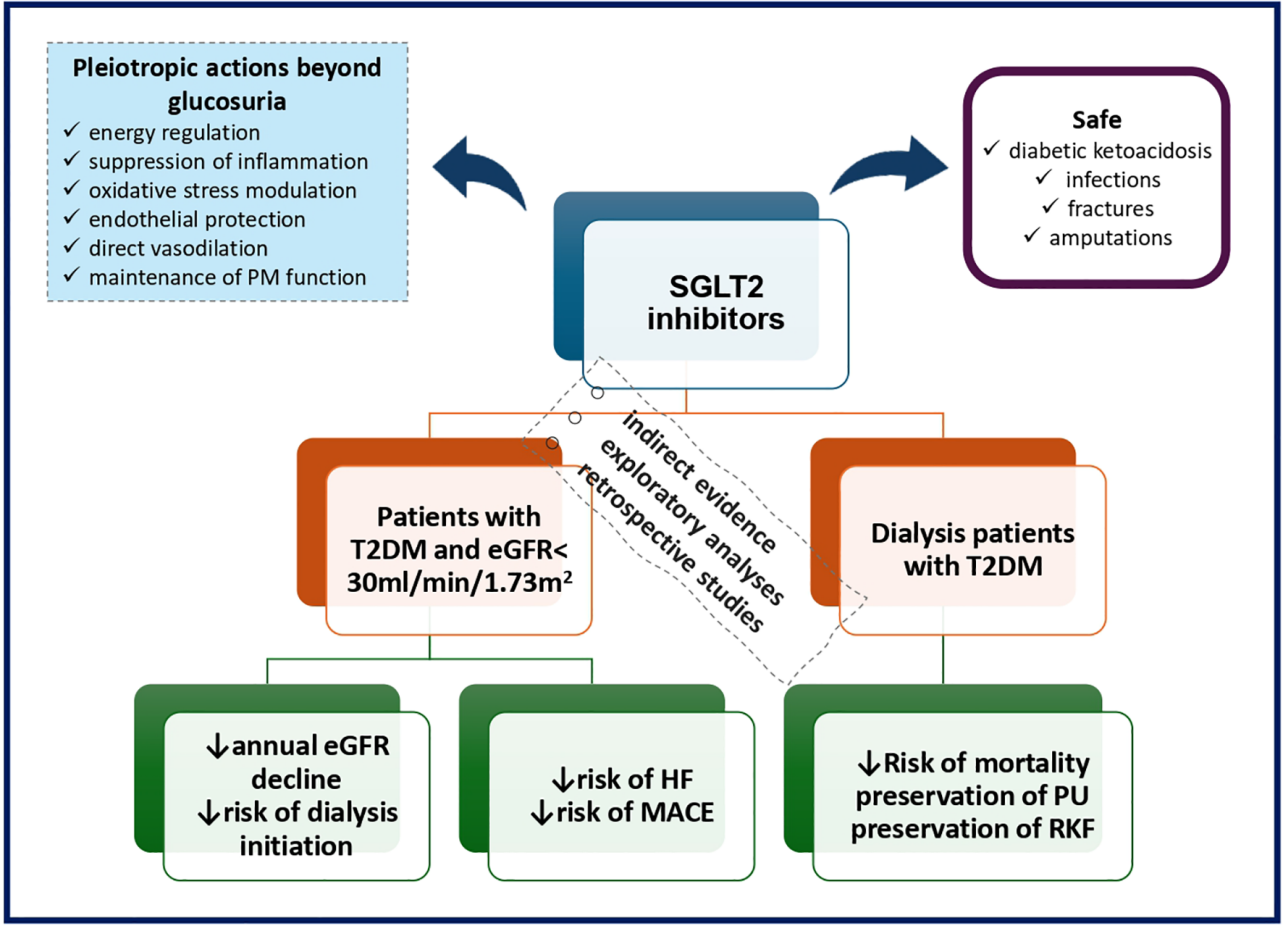
SGLT2 inhibitors in patients with diabetes and advanced CKD. eGFR, estimated glomerular filtration rate; HF, heart failure; MACE, major adverse cardiovascular events; PM, peritoneal membrane; PU, peritoneal ultrafiltration; RKF, residual kidney function; T2DM, type 2 diabetes mellitus.

The aim of this review is to tackle points of contention by presenting current knowledge available on the utility of SGLT2 inhibitors in patients with diabetes and advanced CKD in term of kidney and cardiovascular protection, by addressing potential issues related to safety as well as by providing a foundation based on preliminary data regarding SGLT2 inhibitor benefits in dialysis (**Figure 1**).

## Do the physiological effects of SGLT2 inhibitors persist in the setting of advanced diabetic kidney disease?

When the eGFR falls below the 30 mL/min/1.73 m² cut off point, the question arises whether SGLT2 inhibitors continue to exert kidney and systemic effects despite their limited glucose-lowering efficacy. Although the renal excretion of glucose progressively diminishes in parallel with the declining eGFR, it should be acknowledged that glucosuria persists in minor quantities in advanced CKD (17). A pooled data analysis from five trials involving patients with type 2 diabetes receiving treatment with empagliflozin, showed that the antihypertensive effects were preserved in patients belonging to the lower levels of the eGFR spectrum (18).

Evidence from experimental and mechanistic studies has brought to the forefront the pleiotropic actions of SGLT2 inhibitors related to energy regulation, vascular function, inflammatory pathways and oxidative stress modulation, which remain active even when severe nephron loss has occurred. Even though the nutrient deprivation state is widely considered to be the end result of the glucosuria and of the ensuing loss of calories induced by SGLT2 inhibitors, accumulating evidence supports the activation of nutrient deprivation signals including the sirtuins, AMPK and peroxisome proliferator-activated receptor-γ coactivator-1α (PGC-1α) not only beyond the renal tissue, such as in the liver and the heart, but also in the setting of severely depressed kidney function (19). Accordingly, the effects of SGLT2 inhibitors on the promotion of erythropoietin production have as well been linked to the upregulation of sirtuin 1 in the liver and subsequent direct HIF-2α induction (20).

Point of proof evidence regarding effects of SGLT2 inhibitors extending beyond SGLT2 inhibition has been provided by HF models indicating that SGLT2 deficiency per se does not protect against myocardial dysfunction despite shifts in the myocardial substrate utilization, replicating the established metabolic effects of SGLT2 inhibitors in wild type animals. Thus, SGLT2 knockout mice display improved myocardial remodeling patterns in the setting of pressure overload and reduced infarct size following ischemia and reperfusion injury when treated with empagliflozin (21, 22). Inhibition of the sodium-hydrogen exchanger (NHE) by empagliflozin both in the proximal tubular cells and the cardiac cells, is associated with inhibition of the sodium calcium exchanger expression and calcium/calmodulin-dependent protein kinase II activity, restoration of calcium homeostasis and mitochondrial ATP generation, eventually leading to attenuation of apoptosis and oxidative stress, effects which are particularly valuable in advanced CKD (23–25). Endothelial dysfunction and decreased bioavailability of NO have long been recognized as a hallmark of diabetic nephropathy (26). Several lines of evidence indicate that SGLT2 inhibitors confer protection of the vascular endothelium via complex and intertwining mechanisms. Direct vasodilatory effects of dapagliflozin and empagliflozin have been shown to be mediated by activation of Kv channels and protein kinase A (27, 28). Empagliflozin abolished the effects of uremic sera on cardiac microvascular endothelial cells, including intracellular ROS generation and reduction in NO level, independently of NH1 effects (24). Experimental ex vivo research data indicate that in SGLT2 knockout mice, empagliflozin inhibits the accumulation of lipid droplets in the vascular endothelium as occurs in type 2 DM and as a result downregulates the perpetuation of the inflammatory response and endothelial energy expenditure via pathways involving the NHE1-protein kinase C (PKC)-NOX signaling as well as through others which remain to be elucidated (29). Furthermore, SGLT2 inhibitors appear to promote endothelial progenitor cell mobilization from their bone marrow niche in patients with type 2 diabetes (30, 31).

Suppression of the inflammatory signaling by SGLT2 inhibitors through inhibition of the NF-κB activation and reduced levels of associated pro-inflammatory mediators including TNF-α, MCP-1, IL-1β, and IL-6 have been described both in experimental and clinical models of type 2 diabetes and CKD although the specific receptors and the respective pathways mediating these effects have not been yet determined (32, 33). Accordingly, in SGLT2 knockout mice, dapagliflozin has been shown to reduce the infiltration of the cardiac tissue by pro-inflammatory M1 macrophages in the setting of various noxious stimuli to the myocardium (34). On top of that, treatment with dapagliflozin down-regulated the expression of TNF-a, IL-1 and IL-6 by bone marrow derived macrophages following lipopolysaccharide priming as well as attenuated the expression of fibrosis markers in co-cultures of macrophages with fibroblasts (34). It should be highlighted that macrophages do not express the SGLT2 receptor as shown by bulk RNA-seq and single-cell RNA-seq studies (34). Notwithstanding the dominant opinion that SGLT2 receptor expression is negligible in the immune cells of the acquired immune system, CD4^+^ T cells isolated from healthy individuals when exposed to high glucose concentration, display reduced glucose uptake and IFNγ production following empagliflozin administration (35).

Recent experimental trials from both *in vivo* and *in vitro* models offer novel insights on the protection provided by SGLT2 inhibitors against the morphological and functional changes of the peritoneal membrane in the setting of long-term peritoneal dialysis (PD). Thus, SGLT2 inhibitors appear to reduce peritoneal fibrosis and improve ultrafiltration through the inhibition of fibrotic pathways such as TGF-b/Smad signaling, attenuation of senescence and epithelial to mesenchymal-transition (36–38).

## Do SGLT2 inhibitors confer any kidney related benefits in patients with diabetes and advanced CKD (CKD stage G4 and G5 non-dialysis)?

The kidney protective effects of SGLT-2 inhibitors appear to be maintained in patients with diabetes and advanced CKD and delay the need for dialysis initiation despite the irreversibility of the renal function decline when the most severe stages of the disease are reached. The early landmark cardiovascular outcome trials of SGLT2 inhibitors in type 2 diabetes did not include patients with established CKD. Subsequently, secondary analysis of data from the major CKD SGLT2 trials supports their sustained benefit in patients with type 2 diabetes and advanced CKD. Furthermore, the pivotal trials of SGLT2 inhibitors typically either excluded patients with advanced CKD or included very small numbers, yet secondary analyses from these trials have provided substantial evidence regarding SGLT2 inhibitor related benefits in retarding CKD progression even when the eGFR drops to below the 30 ml/min/1.73 m2 threshold. Thus, a *post hoc* analysis of the Canagliflozin and Renal Outcomes in Type 2 Diabetes and Nephropathy (CREDENCE) trial which enrolled patients with diabetes, urinary albumin-creatinine ratio (UACR) >300 mg/g and eGFR above 30 ml/min per 1.73 m^2^ at screening, indicated that the beneficial effects of canagliflozin on hindering the rate of kidney function decline extended to the 174 patients who displayed eGFR less than 30ml/min/1.73m^2^ at evaluation during randomization in the trial (39). The DAPA- (CKD Dapagliflozin And Prevention of Adverse Outcomes in Chronic Kidney Disease) trial, lowered the enrollment threshold to an eGFR of down to 25 ml/min/1.73 m2 and was designed to primarily assess the impact of SGLT2 inhibitors on renal outcomes in the CKD population regardless of the diabetes status (40). It is noteworthy that nearly 65% of the stage 4 CKD population in the DAPA-CKD trial had type 2 diabetes (40). A prespecified analysis of the DAPA-CKD trial data regarding the 624 patients with stage 4 CKD at baseline and with a mean eGFR of 26.8 ml/min/1.73 m2 showed that dapagliflozin consistently diminished kidney function loss in patients with eGFR less than 30 ml/min/1.75 m2. Accordingly, the chronic eGFR slopes displayed a difference of 1.82 ml/min/1.73 m2 per year ((95% CI, 0.96 to 2.68; P <0.0001) between dapagliflozin and placebo, favoring the former. Furthermore, regarding the composite kidney end point of ≥50% sustained decline in eGFR, kidney failure, or death from kidney disease, there was no evidence of a differential treatment effect between patients with diabetes and those without (40). The EMPA-KIDNEY (Study of Heart and Kidney Protection with Empagliflozin) trial enrolled among others, participants with eGFR of at least 20 but less than 45 ml per minute per 1.73 m2 regardless of the level of albuminuria, with 2282 patients or roughly one third of the study population having an eGFR lower than 30 ml/min/1.73 m2 (41). Slightly above 50% of the study population with stage 4 CKD had diabetes. Accordingly, beyond the second month of the study drug administration, empagliflozin delayed the yearly rate of eGFR impairment by 0.43 ml/min/1.73 m2 (–0.70 to 1.56) in patients with eGFR below 20 ml/min/1.73 m2 and by 0.52 ml/min/1.73 m2 (0.14 to 0.90) in those with eGFR between 20 and 30 ml/min/1.73 m2, corresponding to a 13% (–48 to 21) and 20% (–35 to –5) relative decrease in the chronic eGFR slope (42). It should be highlighted that patients with diabetes were the ones displaying the greatest impact in the eGFR slope differences among the study sub-groups (42). Furthermore, following the EMPA KIDNEY trial completion, patients who were submitted to observation for two additional years had a 23% (HR, 0.77; 95% CI, 0.68-0.87) lower risk of kidney disease progression or death from cardiovascular causes during the active trial and the post-trial follow-up period (43). It should be taken into account that patients belonging to the lower eGFR levels in the EMPKA KIDNEY trial were older and more likely to have diabetes.

The design of the major HF trials of SGLT2 inhibitors did not allow for conducting specific analyses in patients with diabetes and advanced CKD. Still, it should be taken into consideration that the empagliflozin HF trials included patients with advanced CKD since patients with an eGFR above 20 ml/min/1.73 m2 were eligible for inclusion both in the EMPEROR-Reduced (Empagliflozin Outcome Trial in Patients With Chronic Heart Failure With Reduced Ejection Fraction) and in the EMPEROR-Preserved (Empagliflozin Outcome Trial in Patients with Chronic Heart Failure with Preserved Ejection Fraction) trials, with more than half of them having diabetes. Accordingly, the EMPEROR-Reduced trial showed that empagliflozin delayed the annual eGFR drop by 0.63 (-2.31–3.56) ml/min/1.73 m^2^ in patients with stage 4 CKD and that the kidney related benefits of empagliflozin were consistent in the trial regardless of the presence of diabetes (44, 45). The above findings are further reinforced by the results of the EMPEROR-Preserved trial which showed that empagliflozin attenuated the annual eGFR decline by 0.22 (−1.30 to 1.75) compared to placebo (46). These data allow us to infer that empagliflozin improves the kidney outcomes in patients with the cardiorenal syndrome and diabetes.

A recent meta-analysis including 70361 participants from 10 randomized trials, further conferred that SGLT2 inhibitors reduced the risk of CKD progression regardless of the eGFR levels, including patients with diabetes and advanced CKD who are at the highest risk of kidney failure (47). Of note, when treatment effects were examined according to the baseline eGFR levels and diabetes status, significant decreases in the annual rate of eGFR decline were observed in all the subgroups (47). In specific, SGLT2 administration reduced the risk of CKD progression by 34% (HR, 0.66; 95% CI, 0.53-0.82) in the 2262 patients with stage 4 CKD and diabetes who were included in the meta-analysis, an effect which was retained even in the small number of participant with eGFR below 20 ml/min/1.73 m2 (47). Furthermore, the annual decline in eGFR was 1.04 (0.22) ml/min/1.73 m2 in patients with stage 4 CKD and diabetes receiving SGLT2 inhibitors and 2.61 (0.22) ml/min/1.73 m2 in those receiving placebo, accounting for a relative difference of 60% (-76 to -44) (47).

Emerging evidence indicates that SGLT2 inhibitors may offer kidney-related advantages even in patients with stage 5 CKD (**Table 1**). Thus, the most robust data come from a target trial emulation study, including a large population of approximately 48000 patients with type 2 diabetes and eGFR less than 15mL/min/1.73m^2^ from the Taiwan’s National Health Insurance Research Database (NHIRD) (48). During a mean follow-up of 3.1 years, patients initiating SGLT2 inhibitors compared to non-users displayed a decreased risk of starting dialysis both in the intention-to-treat (HR, 0.34 [95% CI, 0.27 to 0.43]) and in the as-treated model (HR, 0.67 [CI, 0.53 to 0.85]) regardless of gender, age or the level of albuminuria (48). A study examining the effectiveness of SGLT2 inhibitors in incident patients with CKD stage 5 and established type 2 diabetes between 2016 and 2020, compared out of a total of 36184 CKD5 patients with type 2 diabetes, 248 patients under treatment with SGLT2 inhibitors with 968 non-users following propensity score matching (49). It should be taken into consideration that most patients were already on SGLT2 inhibitors before reaching CKD stage 5. In agreement with the above, the SGLT2 inhibitor group exhibited a nearly 40% lower risk of dialysis initiation (HR 0.61, 95% CI, 0.47–0.77) (49). Additionally, aretrospective cohort study of adults with stage 5 CKD from the TriNetX global collaborative network database, following propensity score matching of a pool of more than 90000 individuals from 16 countries in Europe, Asia, Australia, North and South America, eventually compared 3465 patients receiving treatment with SGLT2 inhibitors with 3465 patients not using SGLT2 inhibitors, with the vast majority having diabetes in both groups (50). The SGLT2 inhibitors reduced the risk of dialysis initiation by 46% (HR, 0.54; 95% CI, 0.48 – 0.61) during a five-year follow-up (50). A recent retrospective study from France, including 204 patients with stage 4 CKD, with more than 80% having diabetes and who initiated SGLT2 inhibitors approximately 1 year following CKD stage 4 diagnosis, provides real world data regarding the patterns of use of SGLT2 inhibitors (51). Notably, a lower baseline eGFR was a reason for SGLT2 inhibitor discontinuation after 6 months of follow-up and patients who discontinued SGLT2 inhibitors exhibited a significantly sharper decrease in eGFR (−7.3 ± 4.6 ml/min/1.73 m2) compared to those who remained on treatment (−3.8 ± 4.5) (51).

**Table 1:** Summary of the main recent studies on SGLT2 inhibitors in patients with stage 5 CKD.

Study	Design	Inclusion criteria	Main kidney outcomes	Main cardiovascular outcomes	Adverse events
Yen et al., 2024 (48)	Target Trial Emulation Study	T2DM Stage 5 CKD ND <100 years old (N = 47,747)	64% and 33% RR reduction of starting dialysis in the intention-to-treat and the as-treated model respectively	20% and 19% RR reduction of hospitalization for HF and 39% and 43% RR reduction of AMI in the intention-to-treat and the as-treated model respectively	22% and 29% RR reduction of DKA and 20% and 35% RR reduction of AKI in the intention-to-treat and the as-treated model respectively
Huang et al., 2025 (49)	Retrospective cohort Study	T2DM Stage 5 CKD ND ≥20 years old (N = 36,184)	39% RR reduction of new-onset ESKD	12% RR reduction of MACCE (nonsignificant); 11% RR reduction of MACCE-associated mortality (nonsignificant)	3% higher risk of infection-related hospitalization (nonsignificant) 31% RR reduction of infection-associated mortality (nonsignificant)
Anuforo et al., 2025 (50)	Retrospective cohort Study	Stage 5 CKD ND ≥18 and <90 years old (N = 8,940)	36% RR reduction of composite of all-cause mortality, development of ESKD or HF; 40% RR reduction of ESKD (5years follow up)	27%, 35%, 40%, 42%, 40%, 33%, 28% and 36% RR reduction of HF, MI, cardiac arrest, hypertensive urgency, hypertensive crisis, systolic HF, diastolic HF and ventricular tachycardia respectively; 14%, 6%, 12%, 11% and 34% RR reduction of CAD, ischemic stroke, hemorrhagic stroke, angina and ventricular fibrillation respectively (nonsignificant)	31%, 30% and 35% RR reduction of respiratory failure, metabolic encephalopathy or coma and acidosis respectively; 4%, 8% and 21% RR reduction of DKA in T2DM, UTI and lower extremity amputations respectively (nonsignificant)
Wang et al., 2024 (63)	Retrospective cohort Study	T2DM, 18–90 years old who initiated dialysis (N = 1542)	51% RR reduction of needing dialysis in 90 days after dialysis initiation	48% RR reduction of MACE	25%, 3% and 2% higher risk for ketoacidosis, UTI/genital infection and 90-day readmission respectively (nonsignificant); 40%, 5%, 23%, 14% and 29% RR reduction of 3P-MACE, hypoglycemia, dehydration, fracture and below-knee amputation respectively (nonsignificant)
Berrio et al., 2024 (66)	Retrospective observational single-center study	Patients on PD who initiated SGLT2is (N = 16)	Preserved RKF and diuresis	Significant drop in SBP (p=0.003)	1 case of serious hypoglycemia; 1 case of asthenia, general malaise, and recurrent UTI

AKI, acute kidney injury; AMI, acute myocardial infarction; CKD, chronic kidney disease; DKA, Diabetic ketoacidosis; ESKD, end stage kidney disease; HF, heart failure; MACE, non-fatal myocardial infarction, non-fatal ischemic stroke, cardiovascular death/mortality, and hospitalization for angina; RR, relative risk; SGLT2i, sodium glucose cotransporter inhibitors; T2DM, type 2 diabetes mellitus; UTI, urinary tract infections.

## Do the cardiovascular benefits of SGLT2 inhibitors extend to patients with diabetes and advanced CKD (CKD stage G4 and G5 ND)?

SGLT2 inhibitors have revolutionized the management of type 2 diabetes, conferring tremendous and indisputable cardiovascular benefits, particularly in diabetic patients with established or at high risk for atherosclerotic CVD and those with diabetic kidney disease; however, novel, accumulating evidence supports that their cardiovascular benefits extend to patients with diabetes and advanced CKD as well (52). Overall, it should be acknowledged that the SGLT2 trials in CKD involved mixed populations, and no specific analyses were conducted with respect to cardiovascular outcomes in the subgroups of patients according to CKD stages and diabetes status. Accordingly, the *post hoc* analysis of the CREDENCE trial showed no differences with regard to the risk of cardiovascular death, myocardial infarction or stroke and hospitalization for HF in patients with stage 4 CKD compared to patients in earlier CKD stages (39). Even though it should be noted that most patients belonged to the eGFR 20–30 ml/1.73 m2 category and the number of hard cardiovascular outcomes was limited, the results from CREDENCE trial paved the way for continuation of SGLT2 treatment in the setting of advanced CKD. In line with the above, the results of the DAPA CKD trial showed that the relative risk reduction of cardiovascular death or hospital admission for HF and all-cause mortality was consistent both in patients with type 2 diabetes and those without (53). Accordingly, the prespecified analysis of the DAPA-CKD trial data regarding the 624 patients with stage 4 CKD at baseline, showed a 17% reduction in the risk for HF hospitalization or cardiovascular death (HR 0.83; 95% CI, 0.45 to 1.53) in patients treated with dapagliflozin compared to placebo. No significant effects of empagliflozin on specific causes of death or major cardiovascular outcomes were detected in the EMPA KIDNEY trial in the setting of a low number of events which did not allow for a sub-group analysis to be conducted in patients with diabetes and an eGFR less than 30ml/min/1.73m^2^ (41, 43).

Despite the unequivocal evidence provided by the major SGLT2 cardiac trials, with regard to the improved cardiovascular outcomes conferred by SGLT2 inhibitors, including HF exacerbation and cardiovascular death in patients with eGFR less than 30 ml/min/1.73 m2, there are no data available pertaining to patients with diabetes and advanced CKD (44, 46). Pooled analyses of the DAPA-HF (Study to Evaluate the Effect of Dapagliflozin on the Incidence of Worsening Heart Failure or Cardiovascular Death in Patients With Chronic Heart Failure) and the DELIVER (Dapagliflozin Evaluation to Improve the Lives of Patients With Preserved Ejection Fraction Heart Failure) trials which enrolled patients with eGFR levels as low as 30 ml/min/1.73 m2 and 25 mL/min/1.73 m2 respectively, showed that out of a total of 11007 patients randomized in both trials, 347 patients, with 64% of them having diabetes, displayed an impairment of eGFR to levels below 25 mL/min/1.73 m2 at least once during trial follow up (54). The incidence of the composite outcome including hospitalization for HF or urgent HF visits requiring intravenous treatment or cardiovascular death was lower in patients who exhibited an eGFR decline to levels below 25 ml/min/1.73 m2 and who were under treatment with dapagliflozin (HR: 0.53; 95% CI: 0.33-0.83) compared to placebo, an advantage which persisted as well in the 80 patients with sustained eGFR decline on two consecutive measurements of kidney function (54). Furthermore, considering that patients with advanced CKD are at the highest risk for adverse cardiovascular outcomes, the absolute risk reduction with dapagliflozin was more pronounced in patients with eGFR decline below the 25 ml/min/1.73 m2 threshold (54). In line with the above, the EMPEROR-Reduced (Empagliflozin Outcome Trial in Patients With Chronic Heart Failure With Reduced Ejection Fraction) trial, showed that empagliflozin reduced the primary outcome of death from cardiovascular causes or HF hospitalizations in patients with eGFR down to 20 ml/min/1.73 m2 (45). Furthermore, empagliflozin improved the primary composite endpoint of cardiovascular death or first HF hospitalization as well as the separate cardiovascular outcomes in the EMPEROR-Preserved (Empagliflozin Outcome Trial in Patients With Chronic Heart Failure With Preserved Ejection Fraction) regardless of the CKD stage category (46).

Systematic reviews and meta-analyses of the pivotal SGLT2 trials have confirmed the cardiovascular benefits of this class of medications in a large and heterogenous spectrum of patients with advanced CKD (12, 55). Accordingly, a systematic review and meta-analysis of 9 trials and 62329 participants, showed that SGLT2 inhibitors reduced the risk of HF across all the eGFR strata with the most prominent beneficial effects being observed in patients belonging to the eGFR 20–30 ml/min/1.73 m2 subgroup (56). Strikingly, the meta-regression analysis revealed that the HF risk reduction in advanced CKD concerned only patients with diabetes included in the CREDENCE, DECLARE-TIMI 58 (Dapagliflozin and Cardiovascular Outcomes in Type 2 Diabetes), VERTIS (Cardiovascular Outcomes with Ertugliflozin in Type 2 Diabetes), SOLOIST-WHF (Sotagliflozin in Patients with Diabetes and Recent Worsening Heart Failure) and SCORED (Sotagliflozin in Patients with Diabetes and Chronic Kidney Disease) trials (56).

Recent studies, albeit not randomized controlled trials, have shed more light to the cardiovascular effects of SGLT2 inhibitors in patients with diabetes and advanced CKD. Results from a propensity score–matched cohort study including 290 patients with type 2 diabetes and an eGFR less than 30mL/min/1.73m2, receiving treatment with SGLT2 inhibitors, indicate that the use of SGLT2 inhibitors was associated with fewer major adverse cardiovascular events (MACE) in patients with advanced CKD, a benefit appearing to persist even in those with eGFR below 20 ml/min/1.73 m2 (57). A retrospective cohort study of less than 700 patients with type 2 diabetes and CKD showed that the continuation of SGLT2 inhibitors after the eGFR declined to levels below 30ml/min/1.73m^2^, was associated with a significantly lower risk of myocardial infarction during follow-up whereas the risk of HF hospitalization or cardiovascular death did not differ significantly between SGLT2 users and non-users (58). The target trial emulation study by Yen et al. showed that both in the intention-to-treat and in the as-treated model, patients initiating SGLT2 inhibitors displayed a decreased risk of both hospitalization for HF (HR 0.80, 95%CI 0.73-0.86 and HR 0.81, 95%CI 0.73-0.90 respectively) and acute myocardial infarction (HR 0.61, 95%CI 0.52-0.73 and HR 0.57, 95%CI 0.45-0.72 respectively) (48). In agreement with the above, Anuforo et al. showed in their retrospective cohort study that stage 5 CKD patients under treatment with SGLT2 inhibitors displayed a significantly lower 5-year risk of several cardiovascular endpoints including development of HF (HR, 0.81; 95% CI, 0.65 – 0.998, p = 0.048), myocardial infarction (HR 0.70; 95% CI 0.54-0.90, p=0.006), cardiac arrest (HR 0.709; 0.504-0.997, p=0.047), ischemic but not hemorrhagic stroke (HR 0.73; 95% CI 0.54 – 0.98) as well as hypertensive urgency (HR 0.53; 0.38 – 0.955, p<0.0001) and hypertensive crisis (HR 0.54; 0.40 – 073, p<0.0001) (50). Paradoxically, despite the indisputable effects of SGLT2 inhibitors on improving HF related outcomes in patients with diabetes and advanced CKD, real world data indicate that development of HF is an independent predictor of SGLT2 discontinuation in these patients (51).

## Are there potential benefits of SGLT2 inhibitors in patients with diabetes undergoing dialysis - cardiovascular protection and beyond?

The use of SGLT2 inhibitors in patients with diabetes undergoing dialysis remains investigational as several issues need to be clarified, including their pharmacokinetic properties in relation to dialysis modalities, their multisystem pleiotropic effects as well as potential drawbacks. A pharmacokinetic study conducted in a small cohort of hemodialysis and PD patients with and without diabetes, indicated that although dapagliflozin, when tested in a dose of 10 mg daily was not dialyzable, significant drug accumulation did not occur and the peak drug concentrations as well as of its inactive metabolite dapagliflozin-3-O-glucuronide, did not differ significantly when compared to patients with type 2 diabetes but no CKD (59, 60). Consistent with the above, administration of escalating doses of canagliflozin up to 300 mg in dialysis patients did not lead to increased drug exposure compared to healthy subjects, albeit delayed peak drug levels and no removal by the dialysis procedure were observed (61). Despite a paucity of data regarding empagliflozin pharmacokinetics in dialysis, the drug appears to be well tolerated even in patients with kidney failure (17).

Even though the major SGLT2 clinical trials excluded dialysis patients, it is worth mentioning that data from the DAPA CKD trial have shown that the 24 patients who continued dapagliflozin after dialysis initiation experienced fewer deaths compared to the 47 patients who were receiving placebo (62). A retrospective cohort study analyzed data from the electronic health records of 771 patients with type 2 diabetes who were under treatment with SGLT2 inhibitors within 3 months of dialysis initiation in order to evaluate the potential effects of SGLT2 inhibitors on all-cause mortality and cardiovascular outcomes (63). Following propensity score matching, SGLT2 inhibitor users compared to non-users displayed a lower risk of all-cause mortality (adjusted HR = 0.49; 95% CI = 0.34-0.69, p,0.001), corresponding to minus 11% risk difference (95% CI=-0.14- -0.08, p < 0.001) as well as a lower risk of 4- point MACE (adjusted HR = 0.52; 95% CI = 0.36–0.75, p < 0.001), during a mean follow-up period of 2 years (63).

Accumulating data from patients undergoing PD suggests promising results regarding preservation of peritoneal ultrafiltration in the setting of SGLT2 inhibitors’ use. Accordingly, preliminary evidence from studies conducted in small cohorts of PD patients, the majority of whom with type 2 diabetes mellitus, indicates increased daily ultrafiltration together with potential increases in sodium dipping (64, 65). Furthermore, initiation of dapagliflozin and empagliflozin in a small cohort of prevalent PD patients was associated with improved blood pressure control and preserved residual kidney function during a 6-month follow-up (66). Still, the significant limitations of the studies conducted until now should be considered, including their observational nature, the small sample sizes and short follow-up periods. Considering that volume overload is frequent and associated with detrimental outcomes in PD patients as well as the crucial role of residual kidney function and peritoneal ultrafiltration in the regulation of fluid balance in these patients, the need for future research is highly anticipated (67–69).

Ongoing clinical trials in hemodialysis and PD patients shall clarify the impact of SGLT2 inhibitors on diverse outcomes of interest, such as maintenance of residual kidney function and peritoneal membrane characteristics in PD patients with diabetes (EMPIRIC-PD-NCT06483074 trial, CANARY-NCT05715814 trial, EMPOWERED trial-jRCTs051230081) (70). Finally, the DAPA-HD (NCT05179668) is a prospective randomized, controlled, double-blinded phase II trial to examine the effect of the SGLT2 inhibitor dapagliflozin, in comparison with placebo on cardiovascular outcome parameters in hemodialysis patients.

## Are there any potential risks of SGLT2 inhibitors in patients with diabetes and advanced CKD?

Safety issues of SGLT2 inhibitors in the advanced stages of diabetic kidney disease are potentially a subject of concern for prescribing clinicians. Accordingly, even though an acute decline in eGFR during the initiation of all SGLT2 inhibitors is a temporary, reversible event which might also signal nephroprotection in the long term, the question arises whether the eGFR drop would be acceptable in the setting of a significantly reduced renal functional reserve as occurs in advanced CKD. Data from the major SGLT2 clinical trials in advanced CKD provide reassuring results. Accordingly, no significant differences were observed regarding AKI events with canagliflozin compared with placebo in participants with eGFR <30 ml/min/1.73 m2 in the CREDENCE trial (39). The placebo adjusted acute eGFR dip in the EMPA KIDNEY trial was 1.58 ml/min/1.73 m2 (95% CI 1.1-2.06), corresponding to a relative difference of 6% (4–8), thus being similar to the other eGFR subgroups (42). In agreement with the above, the DAPA CKD trial showed that patients with stage 4 CKD receiving dapagliflozin, despite exhibiting a greater decline in eGFR from baseline to two weeks when compared to placebo, the impact of dapagliflozin on the acute eGFR dip was reduced (1.42 versus 2.56 ml/min per 1.73 m^2^ per 2 weeks) when these patients were compared to their counterparts belonging to earlier CKD stages (40). The large target trial emulation study by Yen et al, showed that SGLT2 inhibitor use compared to no use in patients with diabetes and stage 5 CKD was associated with a reduced risk for AKI both in the intention to treat (HR, 0.80 [CI, 0.70 to 0.90]) and in the as treated model (HR, 0.65 [CI, 0.55 to 0.78]) (48). Finally, no increased risk for development of serious acute kidney injury (AKI) was detected in the large meta-analysis by Neun et al. in patients with diabetes and eGFR less than 30 ml/min/1.73 m2, thus providing reassuring evidence in order to support SGLT2 inhibitors administration in patients with advanced CKD (47). Overall, caution is warranted in the setting of advanced diabetic kidney disease, to avoid initiating SGLT2 inhibitors concurrently with diuretics or renin angiotensin system (RAS) blockade due to synergistic effects on renal hemodynamics, whereas patient guidance regarding the “sick day rules”‘ cannot be overemphasized.

About other SGLT inhibitors related outcomes of interest, such as diabetic ketoacidosis, infections, fractures and amputations, the available evidence until now does not indicate an increased risk in patients with diabetes and advanced CKD. Accordingly, it should be noted that in the DAPA CKD trial, although there was a higher rate of serious adverse events in patients with advanced CKD compared to those with stage 2 and 3 CKD, the rate was numerically lower when compared to placebo (40). Regardless of the fact that diabetic ketoacidosis is a well-recognized worrisome albeit rare effect of SGLT2 inhibitors, the available data do not suggest an increased risk in patients with advanced diabetic kidney disease. Furthermore, the limited number of patients in the DAPA CKD trial who continued either the study drug or placebo following dialysis initiation, experienced similar rates of serious adverse events (62). Overall, no cases of diabetic ketoacidosis were reported in the DAPA CKD trial and only 6 events among 3304 participants were reported in the EMPA KIDNEY trial; however, no hints regarding diabetes status or CKD stage were provided (40, 41). The study by Yen et al. showed a lower risk of diabetic ketoacidosis in SGLT2 users with stage 5 CKD both in the intention to treat (HR, 0.78 [CI, 0.71 to 0.85]) and in the as treated analysis (HR, 0.71 [CI, 0.63 to 0.79]) (48). Despite a potentially marginally higher rate of fungal urinary tract infections found in the study by Chan et al, SGLT2 inhibitors do not generally appear to heighten the risk of infections in patients with diabetes and advanced CKD (25, 48, 49, 57, 63). Sex related factors should be taken into account as well, since women with stage 4 CKD and receiving SGLT2 inhibitor therapy appear to be at a significantly higher risk of developing urinary tract infections compared to men (50). Despite particular safety concerns arising in the CANVAS program (Canagliflozin Cardiovascular Assessment Study), the number of fractures or amputations was low in patients with CKD stage 4 both in the active and the placebo arms in the CREDENCE trial (40, 71). Still, it should be taken into account that sub-group analysis in the study by Anuforo et al. indicated a significantly higher risk of lower extremity amputations in men receiving SGLT2 inhibitors compared to non-users, even though the impact of various SGLT2 inhibitors could not be particularized (50).

## Conclusions

The evidence gap regarding the administration of SGLT2 inhibitors in patients with advanced diabetic kidney disease remains despite promising results in terms of nephroprotection and cardioprotection from exploratory analyses of major SGLT2 inhibitor trials as well as emerging data from real world studies. Reassuring data from the until now available studies regarding adverse events related to SGLT2 inhibitors use in advanced diabetic nephropathy address potential hesitancy to achieving large scale drug implementation. Furthermore, considering the established association of lower eGFR with frailty as well as the increasing acknowledgment of frailty in patients with diabetes regardless of age, the beneficial effects of SGLT2 inhibitors appear to be accentuated among this vulnerable patient group (72–74). Yet, the limitations of the current evidence should be recognized, including among others heterogeneities in study design and analysis as well as in the inclusion criteria applied with regard to patients with diabetes and advanced CKD. Future experimental research shall further clarify the pleiotropic and off target effects of SGLT2 inhibitors so as to expand their indications in the setting of diabetes and advanced CKD. Finally, the results of ongoing clinical trials in patients with diabetes and kidney failure as well as in dialysis patients are eagerly awaited. 
